# High level of estrogen on the day before embryo transfer is associated with reduced live birth after frozen embryo transfer: a retrospective cohort study from 14565 cycles

**DOI:** 10.3389/fendo.2026.1900954

**Published:** 2026-07-29

**Authors:** Mi Han, Xiaojuan Wang, Ge Lin, Yuan Li, Fei Gong

**Affiliations:** 1 NHC Key Laboratory of Human Stem Cell and Reproductive Engineering, Xiangya School of Basic Medical Sciences & Furong Laboratory, Central South University, Changsha, China; 2 Clinical Research Center for Reproduction and Genetics in Hunan Province, Reproductive and Genetic Hospital of CITIC-XIANGYA, Changsha, Hunan, China; 3 Hunan Guangxiu Hi-tech Life Technology Co. Ltd, Changsha, China

**Keywords:** endometrial preparation, endometrium, estrogen, freezing-all cycles, frozen embryo transfer

## Abstract

**Background:**

The endometrial preparation of frozen embryo transfer cycle is to synchronize of endometrium and embryo under the condition of exogenous estrogen and progesterone drugs in HRT or follicle monitoring in NC. Estrogen and progesterone are crucial and different hormone levels could imply variations on endometrial preparation. The effect of estrogen and progesterone levels before the embryo transfer on the live birth is still controversial.

**Methods:**

This was a retrospective cohort study involving all infertile women who had undergone freeze-all and first frozen-embryo transfer cycle with HRT or NC in the period from January 2016 to December 2020 at a tertiary care center. Deciles and receiver operating characteristic curves were used to determine the cutoff values for estrogen and progesterone levels on the day before embryo transfer. Multivariate logistic regression was performed to analyze the independent effect of estrogen and progesterone on live birth.

**Results:**

The cutoff value of estrogen level on the day before embryo transfer in the HRT group was 903.10 pg/mL. The lower level of estrogen on the day before embryo transfer in the HRT group corresponded to a higher live birth rate (51.4% vs. 32.9%, *P < 0.001*). The cutoff value of estrogen level on the day before embryo transfer in the NC group was 194.40 pg/mL. The lower level of estrogen in the NC group corresponded to a higher live birth rate (53.0% vs. 48.6%, *P* < 0.001). After adjusting for the possible confounders, estrogen level was the independent influencing factor of the live birth in both HRT and NC, with the odds ratios of 0.56 (0.45, 0.70) and 0.85 (0.76, 0.95), respectively. Progesterone levels had no effect on live birth in either HRT or NC after adjusting for confounders.

**Conclusions:**

In freezing-all cycles, the higher estrogen level on the day before embryo transfer may reduce the live birth rate in HRT and NC.

## Introduction

With the proposal of a freezing-all strategy (1), the proportion of the frozen-embryo transfer (FET) has gradually increased. The European data in 2016 showed that the FET accounted for 44.1% (24.2% in 2007) (2), and the CDC data in the United States in 2018 suggested that the FET accounted for 74.3% (21.4% in 2008) (3). The current focus in the field of the FET is on improving the live birth rate, obstetric outcomes, and offspring safety (4–6). The main indicators influencing these clinical outcomes are endometrial-preparation protocols, luteal-support protocols, embryo-freezing techniques, blastocyst-culture techniques, and embryo-transfer period (7).

At present, the major endometrial preparation protocols are the natural cycle (NC) and hormone replacement therapy cycle (HRT). However, different protocols have different suitable populations (5, 8). The embryo transfer is scheduled by endogenous or exogenous estrogen and progesterone, which imitates the normal menstrual cycle to simulate the endometrial transformation in both NC and HRT. Therefore, the measurement of estrogen and progesterone levels in NC and HRT is particularly important. Previous studies have primarily focused on progesterone levels, but are lacking consistent research conclusions due to different luteal-support protocols, different routes of administration, different drug doses, and different patient characteristics such as age and body mass index (BMI) (9, 10). The difference in pharmacokinetics between the different administration routes results in considerable differences in the peak serum progesterone concentrations, implying an unequal onset of the secretory endometrial transformation between the different progesterone- administration routes (10). However, there is little evidence on estrogen levels, with inconsistent conclusions due to discrepant observation time points and diverse populations (11–13). Therefore, there is several estrogen and progesterone cutoff values to predict the live birth rate in the FET cycle. In the present study, we explored the effect of serum estrogen and progesterone levels on the day before embryo transfer on the live birth rate in patients undergoing freezing-all cycles in the first FET cycle.

## Materials and methods

### Study design and population

A retrospective study was conducted at the Reproductive and Genetic Hospital of CITIC-Xiangya. All infertile women who had undergone freeze-all and first frozen-embryo transfer cycles with HRT or NC in the period from January 2016 to December 2020 were enrolled. Those women whose estrogen or progesterone levels on the day before embryo transfer or live birth outcomes were missing were excluded.

### Endometrial preparation, blood sampling, and thawed embryo transfer

In the NC protocol, ultrasound monitoring and serum hormone measurement were performed from cycle days 12–13 onwards.

1. If the leading follicle reached a diameter of ≥ 12 mm on days 12–13, transvaginal ultrasound was repeated until ovulation. When the diameter of the dominant follicle was ≥ 18–20 mm without ovulation, the endometrium thickness was ≥ 8 mm, and serum progesterone level was < 1 ng/mL, then human chorionic gonadotropin (hCG) 5000–10,000 IU was injected for the final oocyte triggering. Luteal support (10 mg, twice daily; Duphaston, Abbott Biologicals B.V., Abbott Park, IL) was initiated on the day of ovulation and continued for 28 days after embryo transfer if a pregnancy occurred. For luteinized unruptured follicle, the day when the progesterone level was ≥ 1.4 ng/mL after 48 hours of injecting hCG, or the day when the follicular diameter was ≥ 18–20 mm and urine LH was positive and progesterone was ≥ 1.4 ng/mL was assumed to be the ovulation day.
2. If the diameter of the dominant follicle was < 12 mm and progesterone level was < 1 ng/mL on days 12–13, a daily dosage of 75 IU human menopausal gonadotropin (hMG) (Livzon Pharmaceutical Group Inc.) was supplemented to stimulate follicle growth. If there was a dominant follicle after using hMG for 3–5 days, hMG was used continuously until the day of hCG.

In the HRT protocol, oral estradiol tablets (Femoston, Abbott, USA, 2 mg estradiol, twice a day) were commenced on days 3–5 of a natural or progesterone-induced menstrual cycle. The maximum oral estradiol dose was 6 mg. Serum estrogen and progesterone levels and endometrial thickness were monitored after 10 days. Estradiol tablets were additionally administered vaginally before bedtime (Femoston, 2 mg estradiol, every day) in the case of an unsatisfying endometrial thickness. When the endometrial thickness was ≥ 8 mm and progesterone level was < 1 ng/mL, estradiol tablets were administered vaginally until the 10th day after embryo transfer and changed to oral estradiol tablets if hCG was positive. Progesterone vaginal suppositories (200 mg, three times daily; Utrogestan, Besins Healthcare, Paris, France) were given, and oral treatment with dydrogesterone tablets (10 mg, twice daily; Duphaston, Abbott Biologicals B.V.) was initiated on the next day. Late in the morning (8: 00–10: 00) before the day of embryo transfer, plasma samples were drawn for later analysis of estrogen and progesterone concentrations. If clinical pregnancy was confirmed, the dose of estrogen valerate was reduced to 4 mg per day, and the dose was reduced again to 2 mg per day at 45 days after embryo transfer until the drug was discontinued at 55 days after embryo transfer. The combination of Utrogestan and Duphaston was used until 45 days after embryo transfer, and then Duphaston was used alone until 70 days after embryo transfer. If progesterone was > 1 ng/mL on the day of luteal support or endometrial thickness was < 8 mm on the day before the transfer, the HRT cycle was canceled.

All embryos were graded before freezing. On day 3, the embryos were scored using Puissant’s criterion, and blastocysts were graded in line with Gardner and Schoolcraft’s system on days 5/6/7. All embryos were vitrified and thawed using a Kitazato vitrification kit (Kitazato Biopharma, Shizuoka, Japan) in combination with closed High Security Vitrification Straws (Cryo Bio System, France). The embryos were thawed on the day of transfer. The thawed embryos were prioritized based on the best quality before freezing. They were transferred to G2.5 medium and cultured for 2–6 hours. The embryos were considered surviving and suitable for transfer when half or more of the blastomeres recovered or the blastocyst re-expanded.

Two cleavage-stage embryos one good-quality blastocyst or 1–2 non-good blastocysts (depending on the patient’s condition or wishes) were transferred. In the NC group, the day of ovulation was defined as day 0, cleavage-stage embryos were transferred on day 3, and blastocysts were transferred on day 5. In the HRT group, the day of starting progesterone supplementation was considered P + 0, cleavage-stage embryos were transferred on day P + 3, and blastocysts were transferred on day P + 5.

### Outcome measures and definitions of covariates

The primary outcome was live birth, defined as the delivery of a viable infant at 28 gestational weeks or more. The secondary outcomes included intrauterine clinical pregnancy (presence of at least one gestational sac in the uterine cavity on ultrasound at approximately 28 days after embryo transfer), miscarriage (loss of clinical pregnancy before the 28th gestational week), early miscarriage (loss of clinical pregnancy before the 12th gestational week), and intrapartum stillbirth (fetal death during labor after confirmation of the presence of a fetal heartbeat at the onset of labor after 20 weeks of gestation). Miscarriage rates were calculated among clinical pregnancies, and clinical pregnancies included both intrauterine and ectopic pregnancies.

### Statistical analysis

Continuous variables were described as mean ± standard deviation (SD), whereas categorical variables were described with frequency (n) and percentage (%). Deciles and receiver operating characteristic (ROC) curves were used to determine the group cutoff values for estrogen and progesterone levels on the day before embryo transfer. Student’s *t* test or Kruskal–Wallis test was used to compare the continuous variables between the two groups depending on the normality of distribution, and categorical variables were compared using Pearson’s chi-square test or Fisher’s exact test. To analyze the effect of estrogen and progesterone levels on the day before embryo transfer on live birth, multivariate logistic regression analysis was performed to adjust for confounders, including maternal age and BMI, infertility duration, serum estrogen/FSH/LH in the proliferative phase, anti-mullerian hormone (AMH), antral follicle count (AFC), infertility diagnosis (divided into ovulatory disorders, endometrial factors, male factors, tubal factors, and unexplained infertility), stimulation protocol (divided into long protocol, gonadotropin-releasing hormone antagonist protocol, progestin-primed ovarian stimulation, and other protocols [including short protocol and mild stimulation]), insemination methods, using Femoston vaginally, endometrial thickness before embryo transfer, number of embryos transferred, quality of the transferred embryo (divided into no-good-quality embryo, good-quality blastocysts [days 5/6 blastocysts with grade 4BB or higher], and good-quality cleavage-stage embryos [day 3 cleavage-stage embryos with grade 7CI/8C]), and basic diseases (including polycystic ovary syndrome, adenomyosis, moderate to severe intrauterine adhesions, untreated hydrosalpinx, severe uterine malformations, scar uterus, and endometritis). To further explore the effect of estrogen and progesterone level on the day before embryo transfer on live births, subgroup analyses were performed according to the type of embryos transferred and whether Femoston was used vaginally. All the above analyses were performed in HRT and NC. The statistical analyses were performed using IBM SPSS Statistics 24. R software (version 4.2.2) was used to draw the forest plots for subgroup analysis. Significance tests were two-tailed and conducted at the 0.05 significance level.

## Results

### Baseline characteristics

A total of 14,825 patients were initially enrolled. Among them, 260 patients were excluded due to missing estrogen/progesterone levels measured on the day before embryo transfer or missing live birth outcomes, leaving 14,565 patients available for final analysis. Of these, 2484 patients underwent HRT, while 12081 patients underwent NC. In the HRT group, on the day before embryo transfer, the mean estrogen level was 877.03 ± 985.46 pg/mL, the mean progesterone level was 10.06 ± 5.15 ng/mL, and the LBR was 44.6%. In the NC group, on the day before ET, the mean estrogen level was 194.98 ± 347.15 pg/mL, the mean progesterone level was 9.9 ± 5.3 ng/mL, and the LBR was 52.3%. Specific demographic characteristics are shown in **Supplementary Table 1**.

### Association analysis

**Figure 1:**
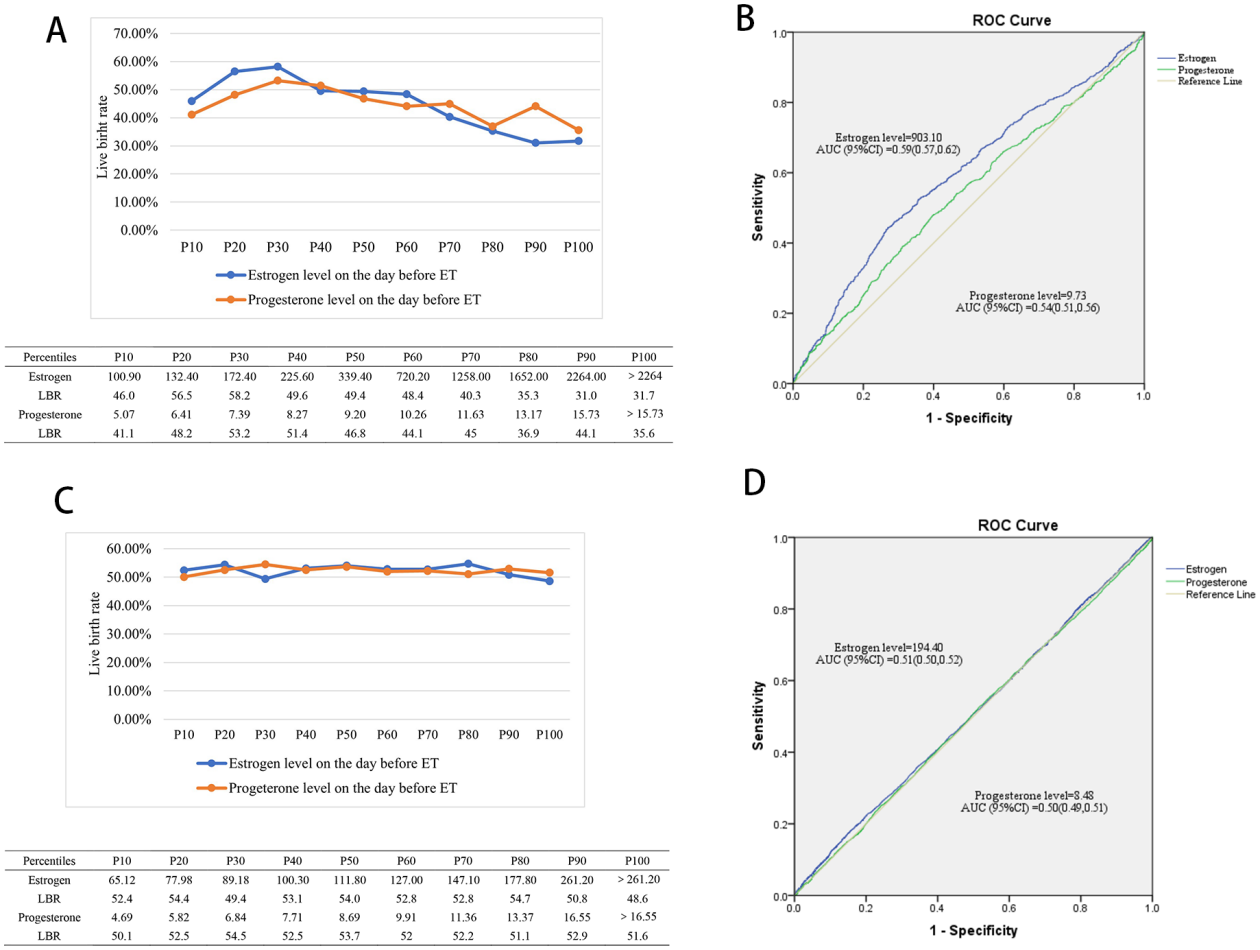
Deciles and receiving operating characteristic (ROC) curve of live birth rate by estrogen and progesterone levels on the day before embryo transfer. **(A)** Deciles in HRT, **(B)** ROC in HRT. **(C)** Deciles in NC, **(D)** ROC in NC. AUC, area under the curve.

Deciles and ROC curves were used to explore the association of estrogen and progesterone levels on the day before embryo transfer with live birth. As shown in **Figure 1A**, the LBR in the HRT group began to decline around the 60th percentile of estrogen and progesterone levels, and the 60th percentiles were close to the cutoff values of ROC curves (shown in **Figure 1B**). Therefore, based on the cutoff value of the ROC curve, the estrogen level before embryo transfer of the HRT group was divided into two groups, namely the group with estrogen < 903.10 pg/mL and the group with estrogen ≥ 903.10 pg/mL. Likewise, the progesterone level before embryo transfer of the HRT group was divided into two groups, namely the group with progesterone < 9.73 ng/mL and the group with progesterone ≥ 9.73 ng/mL. As shown in **Figure 1C**, the LBRs of the NC group were relatively stable at different levels of estrogen and progesterone on the day before embryo transfer. We divided the estrogen levels of the NC group into two groups according to the cutoff value, namely the group with estrogen < 194.40 pg/mL and the group with estrogen ≥ 194.40 pg/mL. Likewise, we divided the progesterone levels of the NC group into two groups, namely the group with progesterone < 8.48 ng/mL and the group with progesterone ≥ 8.48 ng/mL (shown in **Figure 1D**).

Results of univariate analysis showed that the CPR (62.6% vs. 42.7%, *P < 0.001*) and LBR were higher (51.4% vs. 32.9%, *P < 0.001*) and miscarriage rate was lower (19.00% vs. 25.1%, *P = 0.011*) in the group with a low estrogen level (Estrogen < 903.10 pg/mL) compared with the group with a high estrogen level (Estrogen ≥ 903.10 pg/mL) in HRT. Similarly, the CPR (57.9% vs. 52.2%, *P = 0.004*) and LBR were higher (48.3% vs. 40.1%, *P < 0.001*) and miscarriage rate was lower (18.0% vs. 24.4%, *P = 0.003*) in the group with a low progesterone level (Progesterone < 9.73 ng/mL) compared with the group with a high progesterone level (Progesterone ≥ 9.73 ng/mL) in HRT (**Table 1**). In NC, the group with a low estrogen level (Estrogen < 194.4 pg/mL) had higher CPR (62.3% vs. 59.7%, *P = 0.023*) and LBR (53.0% vs. 48.6%, *P < 0.001*) and lower miscarriage rate (16.2% vs. 19.6%, *P = 0.004*) compared with the group with a high estrogen level (Estrogen ≥ 194.4 pg/mL). Pregnancy outcomes were comparable in both progesterone groups (**Table 2**).

**Table 1:** Baseline characteristics and clinical outcomes of different estrogen and progesterone levels on the day before ET in HRT cycles.

Variables	Estrogen <903.10 pg/mL (n=1576)	Estrogen ≥903.10 pg/mL (n=908)	*P*	Progesterone <9.73 ng/mL (n=1380)	Progesterone ≥9.73 ng/mL (n=1104)	*P*
Maternal age (years)	30.57 ± 4.42	32.34 ± 5.04	<0.001	30.57 ± 4.51	32.04 ± 4.88	<0.001
Maternal BMI (kg/m^2^)	22.09 ± 2.81	21.92 ± 2.49	0.318	22.15 ± 2.75	21.87 ± 2.63	0.016
Infertility duration (years)	4.06 ± 2.8	3.94 ± 3.02	0.011	4.05 ± 2.85	3.98 ± 2.93	0.129
Baseline hormonal profile						
FSH (IU/L)	5.76 ± 2.11	6.07 ± 2.54	0.003	5.66 ± 1.93	6.15 ± 2.63	<0.001
LH (IU/L)	5.06 ± 4.29	4.22 ± 3.27	<0.001	5.03 ± 4.59	4.4 ± 2.96	0.008
Estrogen (pg/mL)	40.69 ± 27.90	39.84 ± 26.32	0.459	40.49 ± 28.20	40.25 ± 26.22	0.830
Progesterone (ng/mL)	0.57 ± 1.70	0.51 ± 1.50	0.354	0.55 ± 1.62	0.55 ± 1.64	0.915
AMH (ng/mL)	8.38 ± 6.64	5.93 ± 5.34	<0.001	8.43 ± 6.8	6.31 ± 5.41	<0.001
AFC	31.54 ± 22.27	22.21 ± 16.97	<0.001	31.45 ± 22.81	23.97 ± 17.57	<0.001
Infertility diagnosis			<0.001			<0.001
Ovulatory disorders	666 (42.3)	191 (21)		577 (41.8)	280 (25.4)	
Endometrial factors	44 (2.8)	41 (4.5)		43 (3.1)	42 (3.8)	
Male factor	263 (16.7)	153 (16.9)		202 (14.6)	214 (19.4)	
Tubal factors	569 (36.1)	495 (54.5)		518 (37.5)	546 (49.5)	
Unexplained	34 (2.2)	28 (3.1)		40 (2.9)	22 (2)	
Basic disease						
PCOS	678 (43)	193 (21.3)	<0.001	585 (42.4)	286 (25.9)	<0.001
Adenomyosis	18 (1.1)	14 (1.5)	0.395	21 (1.5)	11 (1)	0.249
Moderate to severe intrauterine Adhesions	232 (14.7)	196 (21.6)	<0.001	207 (15)	221 (20)	0.001
Untreated hydrosalpinx	105 (6.7)	49 (5.4)	0.208	78 (5.7)	76 (6.9)	0.206
Severe uterine malformations	22 (1.4)	8 (0.9)	0.258	11 (0.8)	19 (1.7)	0.036
Scarred uterus	60 (3.8)	104 (11.5)	<0.001	74 (5.4)	90 (8.2)	0.005
Endometritis	136 (8.6)	20 (2.2)	<0.001	105 (7.6)	51 (4.6)	0.002
Stimulation protocol			<0.001			0.010
Long protocol	1109 (70.4)	619 (68.2)		976 (70.7)	752 (68.1)	
Gonadotropin releasing hormone antagonist protocol	235 (14.9)	190 (20.9)		221 (16)	204 (18.5)	
Progestin primed ovarian stimulation protocol	184 (11.7)	61 (6.7)		147 (10.7)	98 (8.9)	
Others (short protocol, mild stimulation, et al)	48 (3)	38 (4.2)		36 (2.6)	50 (4.5)	
Insemination method			0.013			0.024
IVF	1235 (78.4)	712 (78.4)		1070 (77.5)	877 (79.4)	
ICSI	227 (14.4)	154 (17)		207 (15)	174 (15.8)	
IVF+ICSI	114 (7.2)	42 (4.6)		103 (7.5)	53 (4.8)	
Number of oocytes retrieved	15.54 ± 8.31	12.66 ± 7.72	<0.001	15.35 ± 8.25	13.4 ± 8.04	<0.001
Using femoston	568 (36)	866 (95.4)	<0.001	694 (50.3)	740 (67)	<0.001
Estrogen on the day before ET (pg/mL)	249.17 ± 193.91	1966.78 ± 848.2	<0.001	644.38 ± 798.44	1167.83 ± 1112.09	<0.001
Progesterone on the day before ET (ng/mL)	9.16 ± 4.2	11.64 ± 6.16	<0.001	6.85 ± 1.99	14.08 ± 5.06	<0.001
Endometrial thickness before transfer (mm)	11.51 ± 1.61	11.1 ± 1.51	<0.001	11.38 ± 1.61	11.34 ± 1.55	0.591
Number of embryos transferred	1.66 ± 0.47	1.6 ± 0.49	0.002	1.66 ± 0.47	1.6 ± 0.49	0.002
Quality of embryos transferred			<0.001			<0.001
No good-quality embryos	395 (25.1)	268 (29.5)		322 (23.3)	341 (30.9)	
Good-quality blastocysts	364 (23.1)	260 (28.6)		324 (23.5)	300 (27.2)	
Good-quality cleavage stage embryos	817 (51.8)	380 (41.9)		734 (53.2)	463 (41.9)	
Intrauterine clinical pregnancy	987 (62.6)	388 (42.7)	<0.001	799 (57.9)	576 (52.2)	0.004
Miscarriage	190 (19)	100 (25.1)	0.011	146 (18.0)	144 (24.4)	0.003
Early miscarriage	124 (12.4)	66 (16.5)	0.040	97 (11.9)	93 (15.8)	0.038
Live birth	810 (51.4)	299 (32.9)	<0.001	666 (48.3)	443 (40.1)	<0.001
Intrapartum stillbirth	2 (0.2)	0 (0)	0.372	0 (0)	2 (0.3)	0.097

Date are presented as the mean ± standard deviation or n (%).

Difference between the groups were analyzed by the Mann-Whitney U-test or chi-squared test.

**Table 2:** Baseline characteristics and clinical outcomes of different estrogen and progesterone levels on the day before ET in NC cycles.

Variables	Estrogen <194.40 pg/mL (n=10066)	Estrogen ≥194.40 pg/mL (n=2015)	*P*	Progesterone <8.48 ng/mL (n=5811)	Progesterone ≥8.48 ng/mL (n=6270)	*P*
Maternal age (years)	31.83 ± 4.73	31.46 ± 4.63	<0.001	31.8 ± 4.86	31.74 ± 4.57	0.896
Maternal BMI (kg/m^2^)	21.72 ± 2.53	21.24 ± 2.28	<0.001	22.01 ± 2.57	21.3 ± 2.38	<0.001
Infertility duration (years)	3.97 ± 3	3.71 ± 2.64	0.016	4.06 ± 3.1	3.8 ± 2.78	0.001
Baseline hormonal profile						
FSH (IU/L)	6.22 ± 2.15	5.95 ± 1.92	<0.001	6.23 ± 2.15	6.13 ± 2.08	0.071
LH (IU/L)	3.97 ± 3.06	4.23 ± 3.29	0.001	3.94 ± 3.5	4.09 ± 2.69	<0.001
Estrogen (pg/mL)	41.17 ± 27.63	43.24 ± 29.97	0.003	39.75 ± 25.88	43.15 ± 29.82	<0.001
Progesterone (ng/mL)	0.66 ± 2.00	0.66 ± 1.97	0.869	0.61 ± 1.86	0.71 ± 2.11	0.011
AMH (ng/mL)	4.95 ± 4.01	5.69 ± 4.34	<0.001	4.93 ± 3.99	5.21 ± 4.14	<0.001
AFC	20.17 ± 11.98	21.89 ± 12.18	<0.001	20.12 ± 11.99	20.77 ± 12.06	0.001
Infertility diagnosis			<0.001			<0.001
Ovulatory disorders	986 (9.8)	322 (16)		583 (10)	725 (11.6)	
Endometrial factors	427 (4.2)	77 (3.8)		254 (4.4)	250 (4)	
Male factor	3107 (30.9)	565 (28)		1681 (28.9)	1991 (31.8)	
Tubal factors	5125 (50.9)	971 (48.2)		3056 (52.6)	3040 (48.5)	
Unexplained	421 (4.2)	80 (4)		237 (4.1)	264 (4.2)	
Basic disease						
PCOS	1013 (10.1)	326 (16.2)	<0.001	598 (10.3)	741 (11.8)	0.008
Adenomyosis	277 (2.8)	74 (3.7)	0.025	166 (2.9)	185 (3)	0.759
Moderate to severe intrauterine Adhesions	1118 (11.1)	412 (20.4)	<0.001	460 (7.9)	1070 (17.1)	<0.001
Untreated hydrosalpinx	849 (8.4)	140 (6.9)	0.026	614 (10.6)	375 (6)	<0.001
Severe uterine malformations	74 (0.7)	34 (1.7)	<0.001	44 (0.8)	64 (1)	0.124
Scarred uterus	503 (5)	105 (5.2)	0.688	304 (5.2)	304 (4.8)	0.336
Endometritis	1725 (17.1)	292 (14.5)	0.004	907 (15.6)	1110 (17.7)	0.002
Stimulation protocol			<0.001			<0.001
Long protocol	6771 (67.3)	1329 (66)		3957 (68.1)	4143 (66.1)	
Gonadotropin releasing hormone antagonist protocol	1421 (14.1)	220 (10.9)		877 (15.1)	764 (12.2)	
Progestin primed ovarian stimulation protocol	1615 (16)	425 (21.1)		803 (13.8)	1237 (19.7)	
Others (short protocol, mild stimulation, et al)	259 (2.6)	41 (2)		174 (3)	126 (2)	
Insemination method			0.159			0.472
IVF	7499 (74.5)	1542 (76.5)		4358 (75)	4683 (74.7)	
ICSI	1773 (17.6)	328 (16.3)		1019 (17.5)	1082 (17.3)	
IVF+ICSI	794 (7.9)	145 (7.2)		434 (7.5)	505 (8.1)	
Number of oocytes retrieved	13.79 ± 7.38	13.8 ± 7.06	0.815	13.74 ± 7.58	13.84 ± 7.09	0.081
Using femoston	58 (0.6)	339 (16.8)	<0.001	220 (3.8)	177 (2.8)	0.003
Estrogen on the day before ET (pg/mL)	106.65 ± 36.37	636.27 ± 694.55	<0.001	173.31 ± 347.56	215.07 ± 345.58	<0.001
Progesterone on the day before ET (ng/mL)	9.29 ± 4.42	12.96 ± 7.73	<0.001	6.03 ± 1.64	13.49 ± 4.98	<0.001
Endometrial thickness before transfer (mm)	11.97 ± 1.84	11.76 ± 1.9	<0.001	12.01 ± 1.85	11.86 ± 1.85	<0.001
Number of embryos transferred	1.72 ± 0.45	1.57 ± 0.5	<0.001	1.82 ± 0.38	1.57 ± 0.49	<0.001
Quality of embryos transferred			<0.001			<0.001
No good-quality embryos	2272 (22.6)	665 (33)		911 (15.7)	2026 (32.3)	
Good-quality blastocysts	1782 (17.7)	608 (30.2)		627 (10.8)	1763 (28.1)	
Good-quality cleavage stage embryos	6012 (59.7)	742 (36.8)		4273 (73.5)	2481 (39.6)	
Intrauterine clinical pregnancy	6275 (62.3)	1202 (59.7)	0.023	3619 (62.3)	3858 (61.5)	0.398
Miscarriage	1035 (16.2)	239 (19.6)	0.004	608 (16.5)	666 (17)	0.571
Early miscarriage	714 (11.2)	163 (13.4)	0.029	393 (10.7)	484 (12.4)	0.022
Live birth	5339 (53)	980 (48.6)	<0.001	3071 (52.8)	3248 (51.8)	0.250
Intrapartum stillbirth	3 (0.05)	0 (0)	0.449	1 (0.03)	2 (0.05)	0.600

Date are presented as the mean ± standard deviation or n (%).

Difference between the groups were analyzed by the Mann-Whitney U-test or chi-squared test.

After adjusting for possible confounding factors by multivariate logistic regression, we found that the estrogen levels on the day before embryo transfer in both HRT and NC were the independent influencing factors of live birth, with an odds ratio (OR) of 0.56 (0.45, 0.70) and 0.85 (0.76, 0.95), respectively. Progesterone levels had no effect on live birth in either HRT or NC after adjusting for confounders (**Supplementary Table 1**; **Table 3**).

**Table 3:** Multivariable logistic regression analysis of the effect of estrogen and progesterone level on the day before ET on live birth.

Variables	HRT		NC	
	Crude OR (95% CI)	Adjusted OR (95% CI)	Crude OR (95% CI)	Adjusted OR (95% CI)
Estrogen (High vs Low, Low=ref)	0.46 (0.39, 0.55)	0.56 (0.45, 0.7)	0.84 (0.76, 0.92)	0.85 (0.76, 0.95)
Progesterone (High vs Low, Low=ref)	0.72 (0.61, 0.84)	0.94 (0.79, 1.12)	0.96 (0.89, 1.03)	0.98 (0.90, 1.07)

### Subgroup analysis

**Figure 2:**
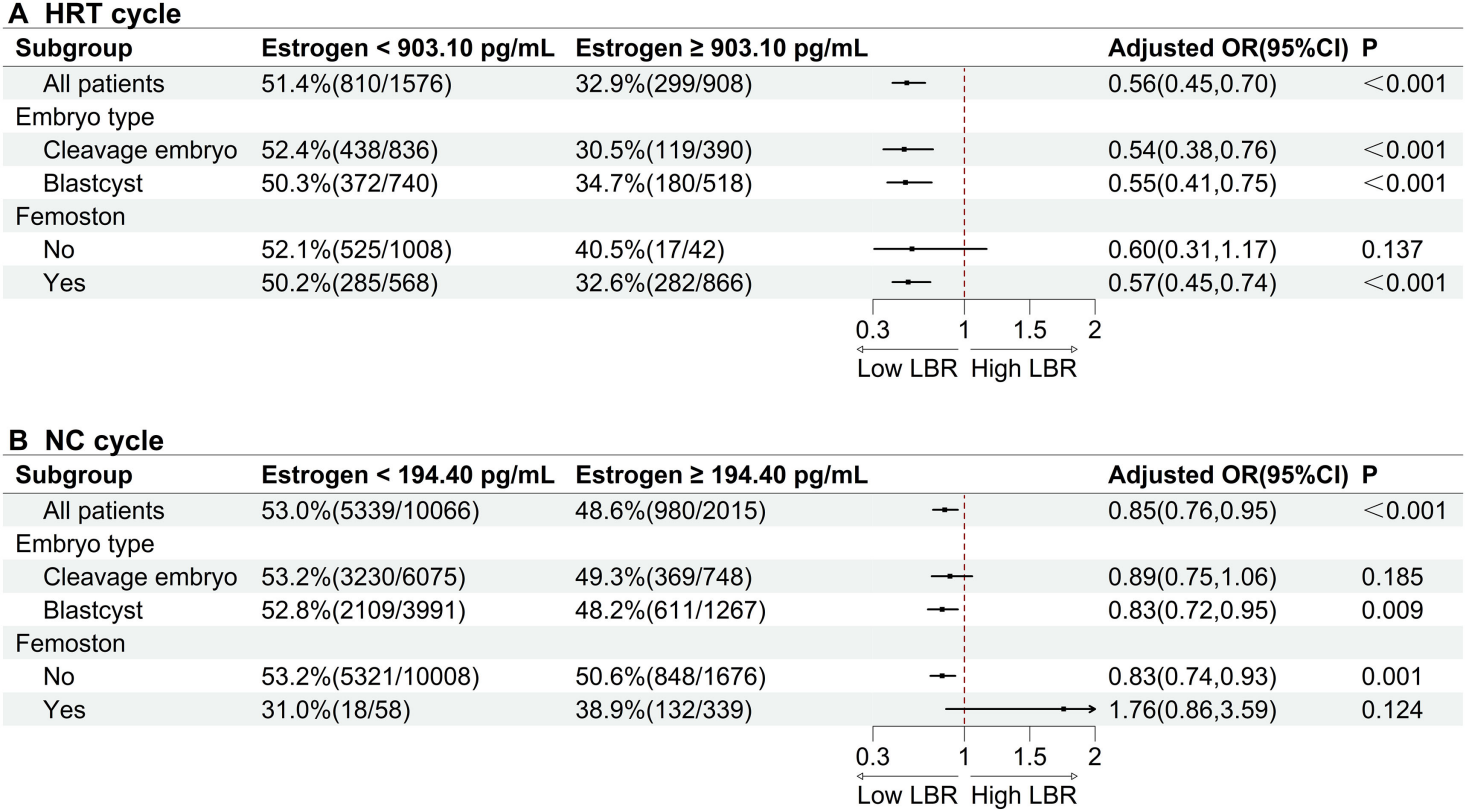
Subgroup analysis of live birth rate in HRT and NC.

Considering that cleavage embryos and blastocysts are transferred at different time points, the corresponding estrogen levels may differ on the day before transfer. In addition, Femoston administered vaginally during treatment has a large impact on estrogen levels. Thus, to further explore the effect of estrogen on live birth, we conducted subgroup analyses based on the type of embryo transferred and whether or not Femoston was used. The results of the subgroup analysis showed that low estrogen levels had higher LBRs in the three subgroups of blastocyst transfer and cleavage-embryo transfer and Femoston administered vaginally in HRT, with the adjusted Odds Ratios (aORs) of 0.55 (0.41, 0.75), 0.54 (0.38, 0.76), and 0.57 (0.45, 0.74), respectively. In NC, estrogen level affected live birth only in the subgroups of blastocyst transfer and without Femoston administered vaginally, with ORs of 0.83 (0.72, 0.95) and 0.83 (0.74, 0.93), respectively (**Figure 2**).

## Discussion

The study found that estrogen in the luteal phase (the day before embryo transfer) significantly affected the live birth rate in the FET with NC or HRT. Higher estrogen levels were associated with lower live birth rates. The estrogen level before embryo transfer was an independent factor affecting the live birth rate after adjusting for the confounders by multivariate logistic regression. Progesterone levels had no effect on live birth in either HRT or NC after adjusting for confounders.

Previous studies have suggested that estrogen overexpression in early pregnancy can induce cell apoptosis and inhibit the proliferation and invasion of trophoblasts (14). Supraphysiologic estrogen levels have been shown to alter the expression of genes and implantation factors in the perimplantation endometrium and affect the pregnancy outcome (15). Previously published studies have disputed the controversial predictive value of estrogen levels in HRT for clinical outcomes (12, 13, 16–19). The inconsistent conclusions mainly come from the following reasons: First, the research populations are not uniform, and the heterogeneity of the population may affect the reliability of the conclusions (12, 13, 16–19). Second, the estrogen detection time differs among different studies. Most studies have explored the estrogen level before progesterone initiation, where the estrogen level may not sufficiently reflect the state of the implantation period, and the prediction value for clinical outcomes is relatively limited (12, 13, 16, 18,). Finally, exogenous estrogen drugs used by study subjects are relatively single dosage and route of administration, so the range of estrogen level fluctuation is limited (12, 13, 16–18), except for one study where the fluctuation of estrogen is more than 2400 pg/mL (20). Moreover, the difference in hormone levels may come from the difference in population demographics (such as the influence of BMI) under the relatively uniform state of hormone dosage and duration (9). The study focused on the estrogen level on the day before embryo transfer, which is relatively close to the embryo transfer period, so it may have greater predictive value for clinical outcomes. Our large sample data contained the estrogen status under different HRT administration routes. Therefore, a larger estrogen concentration range (P5–P95, 104–3190 pg/mL) was used for analysis to obtain real-world data. Our results suggested that serum estrogen level in the HRT exceeding 903.10 pg/mL significantly reduces the live birth rate. Through multivariate logistic regression analysis and subgroup analysis, we found that the high estrogen level was mainly due to the vaginal administration of Femoston. The clinical use of Femoston for vaginal administration comes from the habits of doctors, the demands of patients, and the endometrial status of patients. After adjusting the population demographics for basic diseases such as moderate to severe intrauterine adhesions, our data showed that estrogen was still an independent factor in predicting live birth, indicating that the reason for the difference in estrogen level may be the way of estrogen drug administration with vaginal administration. Femoston is directly absorbed locally in the vagina, without the first-pass effect of oral drug in the liver. Therefore, estrogen drug concentration in the serum is significantly increased, even reaching 4800 pg/mL, according to previous literature (20).

High estrogen levels are associated with many drawbacks, such as a higher risk of vascular disease (21, 22), lower live birth rate, and higher risk of adverse outcomes in the offspring (23–25). Previous literature suggests that the supraphysiologic level of estrogen in the fresh cycle is not conducive to pregnancy outcome, partly due to the downregulation of implantation-related genes and the reduction of endometrial receptivity (15). One of the reasons for the freezing-all trend is that the level of estrogen and progesterone in the FET cycle is more consistent with the physiological level, which may help improve the pregnancy outcome. Therefore, the high estrogen level in the HRT cycle may be detrimental to the pregnancy outcome, which should be controlled by reducing the time of vaginal administration of estrogen as much as possible. Based on the current data, we consider that physician prescribing habits may have led to the use of vaginal Femoston, resulting in high estrogen levels before embryo transfer and consequently adversely affecting live birth outcomes. Future prospective randomized controlled trials are required to further corroborate these conclusions.

Current research focuses on the influence of the length of the follicular phase and the duration of relatively high estrogen levels in the NC (11, 26). The study suggested that estrogen levels exceeding 194.4 pg/mL were unfavorable to the pregnancy outcome. Moreover, the cutoff value of estrogen level was only valid in the blastocyst-transfer subgroup and the non-Femoston subgroup. The main reason may be that the estrogen level reaches its peak before ovulation, decreases during ovulation, and gradually rises after ovulation, with the estrogen level on the day before the cleavage-embryo transfer not rising to 194.4 pg/mL. However, the estrogen level in the vaginal administration cycle of Femoston was greater than 194.4 pg/mL. Therefore, there was no statistically significant difference between these two subgroups. However, the main reason for the different estrogen levels (P5–P95, 55–337 pg/mL) in the NC group was that this study included true NC and mild-ovarian stimulation cycles with the use of low-dose hMG. A mild ovarian stimulation cycle can help reduce the risk of canceling the cycle due to follicular dysplasia (27). Previous studies have confirmed that the mild stimulation cycle does not reduce the clinical outcome of the frozen-embryo cycle (27). However, the use of hMG in ovarian stimulation also increases the risk of developing multiple follicles, which is similar to the effect of supraphysiologic hormone level in the NC and is not conducive to the pregnancy outcome. Therefore, it is also necessary to control the dosage of hMG drugs to reduce the risk of multiple follicular development and control the estrogen level in the luteal phase. It is important to recognize that the origin and clinical meaning of elevated estradiol differ fundamentally between HRT and NC protocols. In HRT cycles, elevated estradiol primarily reflects exogenous estrogen administration particularly vaginal Femoston, which bypasses first-pass hepatic metabolism and results in significantly higher serum concentrations. In NC cycles, elevated estradiol reflects endogenous production from developing follicles, often augmented by hMG stimulation in cases of follicular dysplasia. Despite these different origins, elevated estradiol in both settings is associated with a state of supraphysiologic hormonal exposure that may adversely affect endometrial receptivity through shared mechanisms, including altered gene expression and impaired trophoblast invasion.

Many studies have focused on the effect of progesterone levels during the luteal phase on pregnancy outcomes, and have explored the potential benefits of individualized supplementation of progesterone (9, 10). However, it is still controversial to detect progesterone levels in the luteal phase. The plasma concentration of progesterone is differential because of different routes of administration of progesterone, different doses of administration, and population heterogeneity (such as age and BMI) (9, 10). However, the use time of micronized progesterone for vaginal administration is relatively inconsistent (three times a day but not Q8h). Meanwhile, dydrogesterone is used in both HRT and NC, which the progesterone level in plasma cannot be detected. The additional supplement of dydrogesterone may rescue the relatively low level of progesterone (28). Therefore, progesterone levels measured in serum had no significant association with live birth after adjusting for confounders in the context of the combined luteal support regimen (vaginal micronized progesterone plus oral dydrogesterone).

The strength of this study is the large sample size of the first frozen-embryo transfer patients in the freezing-all cycle, which is conducive to the comparability of the population. Meanwhile, the different routes of estrogen administration and different types of NC (hMG ovarian stimulation and true NC) can better reflect the different hormone level ranges in the real world. However, the limitations of the retrospective study still exist. The use of Femoston in the HRT depends on the habits of doctors, the demands of patients, and the endometrial status of patients. It is helpful to adjust the population to obtain relatively reliable statistical results as far as possible by the multiple regression analysis. However, the diversity of the population cannot be completely ruled out. Admittedly, reducing vaginal estradiol exposure in HRT cycles or hMG dosage in NC cycles may theoretically increase the risk of cycle cancellation due to inadequate endometrial thickness or follicular maturation. However, under our centre’s routine monitoring, the respective cancellation rates were 1.77% and 1.31%, which are considered clinically acceptable. Nonetheless, clinical decisions should be individualised; for patients with a history of thin endometrium or ovulatory disorders, blanket dose reduction may not be appropriate. Future prospective studies are warranted to define optimal strategies that minimise luteal-phase estradiol elevation without substantially increasing cancellation rates.

## Conclusions

In HRT cycles, elevated estradiol levels on the day before embryo transfer are associated with reduced live birth rates, largely driven by vaginal estradiol administration. In NC cycles, a similar but more modest association exists, particularly in cycles with hMG stimulation. These findings suggest that clinicians should consider protocol-specific strategies: minimizing unnecessary vaginal estradiol exposure in HRT and controlling hMG dosage in NC to prevent supraphysiologic estradiol levels.

## Data Availability

The raw data supporting the conclusions of this article will be made available by the authors, without undue reservation.
