# Supplementary material for: High level of estrogen on the day before embryo transfer is associated with reduced live birth after frozen embryo transfer: a retrospective cohort study from 14565 cycles
Source: Front Endocrinol (Lausanne). 2026 Jul 29;17:1900954. doi: 10.3389/fendo.2026.1900954 (PMC13461423; doi:10.3389/fendo.2026.1900954)
Supplement: Supplementary file 1 [file Table1.docx]

**Table S1 Baseline characteristics and clinical outcomes of HRT and NC cycles**

| Variables | HRT （n=2484） | NC （n=12081） | P |
| --- | --- | --- | --- |
| Maternal age (years) | 31.22±4.73 | 31.77±4.71 | 0.000 |
| Maternal BMI (kg/m^2^) | 22.03±2.7 | 21.64±2.5 | 0.000 |
| Infertility duration (years) | 4.02±2.88 | 3.93±2.94 | 0.012 |
| Baseline hormonal profile |  |  |  |
| FSH (IU/L) | 5.87±2.28 | 6.18±2.11 | 0.000 |
| LH (IU/L) | 4.75±3.96 | 4.02±3.1 | 0.000 |
| Estrogen (pg/ml) | 40.38±27.33 | 41.51±28.04 | 0.062 |
| Progesterone (ng/ml) | 0.55±1.63 | 0.66±1.99 | 0.003 |
| AMH (ng/mL) | 7.49±6.31 | 5.07±4.07 | 0.000 |
| AFC | 28.13±20.98 | 20.46±12.03 | 0.000 |
| Infertility diagnosis |  |  | 0.000 |
| Ovulatory disorders | 857(34.5) | 1308(10.8) |  |
| Endometrial factors | 85(3.4) | 504(4.2) |  |
| Male factor | 416(16.7) | 3672(30.4) |  |
| Tubal factors | 1064(42.8) | 6096(50.5) |  |
| Unexplained | 62(2.5) | 501(4.1) |  |
| Basic disease |  |  |  |
| PCOS | 871(35.1) | 1339(11.1) | 0.000 |
| Adenomyosis | 32(1.3) | 351(2.9) | 0.000 |
| Moderate to severe intrauterine Adhesions | 428(17.2) | 1530(12.7) | 0.000 |
| Untreated hydrosalpinx | 154(6.2) | 989(8.2) | 0.001 |
| Severe uterine malformations | 30(1.2) | 108(0.9) | 0.142 |
| Scarred uterus | 164(6.6) | 608(5) | 0.001 |
| Endometritis | 156(6.3) | 2017(16.7) | 0.000 |
| Stimulation protocol |  |  | 0.000 |
| Long protocol | 1728(69.6) | 8100(67) |  |
| Gonadotropin releasing hormone antagonist protocol | 425(17.1) | 1641(13.6) |  |
| Progestin primed ovarian stimulation protocol | 245(9.9) | 2040(16.9) |  |
| Others (short protocol, mild stimulation, et al) | 86(3.5) | 300(2.5) |  |
| Insemination method |  |  | 0.001 |
| IVF | 1947(78.4) | 9041(74.8) |  |
| ICSI | 381(15.3) | 2101(17.4) |  |
| IVF+ICSI | 156(6.3) | 939(7.8) |  |
| Number of oocytes retrieved | 14.49±8.21 | 13.79±7.33 | 0.005 |
| Using femoston | 1434(57.7) | 397(3.3) | 0.000 |
| Estrogen on the day before ET (pg/ml) | 877.03±985.46 | 194.98±347.15 | 0.000 |
| Progesterone on the day before ET (ng/ml) | 10.06±5.15 | 9.9±5.3 | 0.000 |
| Endometrial thickness before transfer (mm) | 11.36±1.58 | 11.94±1.85 | 0.000 |
| Number of embryos transferred | 1.63±0.48 | 1.69±0.46 | 0.000 |
| Quality of embryos transferred |  |  | 0.000 |
| No good-quality embryos | 663(26.7) | 2937(24.3) |  |
| Good-quality blastocysts | 624(25.1) | 2390(19.8) |  |
| Good-quality cleavage stage embryos | 1197(48.2) | 6754(55.9) |  |
| Clinical pregnancy | 1375(55.4) | 7477(61.9) | 0.000 |
| Miscarriage | 290(20.7) | 1274(16.8) | 0.000 |
| Early miscarriage | 190(13.6) | 877(11.5) | 0.032 |
| Live birth | 1109(44.6) | 6319(52.3) | 0.000 |
| Stillbirth | 2(0.14) | 3(0.04) | 0.132 |

Date are presented as the mean ± standard deviation or n (%).

Difference between the groups were analyzed by the Mann-Whitney U-test or chi-squared test.

**Table S2 Multivariable logistic regression analysis for live birth outcomes in HRT cycles and NC cycles.**

|  | HRT | | |  | NC | | |
| --- | --- | --- | --- | --- | --- | --- | --- |
| Variable | Estimate | P | OR95%CI |  | Estimate | P | OR95%CI |
| Maternal age (years) |  |  |  |  |  |  |  |
| <36 | ref |  |  |  | ref |  |  |
| 37-42 | -0.885 | 0.000 | 0.41(0.3,0.57) |  | -0.845 | 0.000 | 0.43(0.38,0.49) |
| ≥43 | -1.518 | 0.007 | 0.22(0.06,0.59) |  | -2.651 | 0.000 | 0.07(0.04,0.12) |
| Maternal BMI (kg/m^2^) |  |  |  |  |  |  |  |
| <18.5 | ref |  |  |  | ref |  |  |
| 18.5-24.9 | 0.008 | 0.959 | 1.01(0.75,1.35) |  | 0.068 | 0.281 | 1.07(0.95,1.21) |
| 25-27.9 | 0.123 | 0.523 | 1.13(0.78,1.65) |  | 0.097 | 0.289 | 1.1(0.92,1.32) |
| ≥28 | 0.092 | 0.762 | 1.1(0.6,1.99) |  | 0.061 | 0.713 | 1.06(0.77,1.47) |
| Infertility diagnosis |  |  |  |  |  |  |  |
| Ovulatory disorders | ref |  |  |  | ref |  |  |
| Tubal factors | 0.030 | 0.961 | 1.03(0.3,3.51) |  | -0.568 | 0.099 | 0.57(0.29,1.12) |
| Endometrial factors | 1.175 | 0.187 | 3.24(0.57,19.3) |  | -0.585 | 0.174 | 0.56(0.24,1.3) |
| Male factor | -0.073 | 0.915 | 0.93(0.24,3.58) |  | -0.403 | 0.261 | 0.67(0.33,1.35) |
| Unexplained | 0.060 | 0.930 | 1.06(0.27,4.14) |  | -0.499 | 0.186 | 0.61(0.29,1.27) |
| Insemination method |  |  |  |  |  |  |  |
| IVF | ref |  |  |  | ref |  |  |
| ICSI | 0.038 | 0.764 | 1.04(0.81,1.33) |  | -0.081 | 0.139 | 0.92(0.83,1.03) |
| IVF+ICSI | -0.089 | 0.615 | 0.91(0.65,1.29) |  | -0.016 | 0.822 | 0.98(0.86,1.13) |
| Stimulation protocol |  |  |  |  |  |  |  |
| Long protocol | ref |  |  |  | ref |  |  |
| Antagonist protocol | -0.101 | 0.440 | 0.9(0.7,1.17) |  | -0.124 | 0.056 | 0.88(0.78,1) |
| Progestin primed ovarian  stimulation protocol | -0.198 | 0.205 | 0.82(0.6,1.11) |  | -0.068 | 0.241 | 0.93(0.83,1.05) |
| Others (short protocol, mild stimulation, et al) | 0.472 | 0.115 | 1.6(0.89,2.87) |  | -0.218 | 0.134 | 0.8(0.6,1.07) |
| Number of oocytes retrieved |  |  |  |  |  |  |  |
| <4 | ref |  |  |  | ref |  |  |
| 4-9 | 0.387 | 0.120 | 1.47(0.91,2.43) |  | 0.166 | 0.114 | 1.18(0.96,1.45) |
| ≥10 | 0.647 | 0.012 | 1.91(1.16,3.2) |  | 0.390 | 0.000 | 1.48(1.2,1.83) |
| Using Femoston (Yes vs No, No=ref) | 0.023 | 0.832 | 1.02(0.83,1.27) |  | -0.259 | 0.031 | 0.77(0.61,0.98) |
| Quality of embryos transferred |  |  |  |  |  |  |  |
| No good-quality embryos | ref |  |  |  | ref |  |  |
| Good-quality blastocysts | 0.645 | 0.000 | 1.91(1.44,2.52) |  | 0.511 | 0.000 | 1.67(1.47,1.89) |
| Good-quality cleavage stage embryos | 0.131 | 0.276 | 1.14(0.9,1.44) |  | -0.102 | 0.074 | 0.9(0.81,1.01) |
| Infertility duration (years) | 0.007 | 0.665 | 1.01(0.98,1.04) |  | -0.014 | 0.036 | 0.99(0.97,1) |
| AMH (ng/mL) | 0.000 | 0.989 | 1(0.98,1.02) |  | -0.009 | 0.167 | 0.99(0.98,1) |
| FSH (IU/L) | -0.011 | 0.646 | 0.99(0.94,1.04) |  | -0.007 | 0.532 | 0.99(0.97,1.01) |
| LH (IU/L) | 0.018 | 0.161 | 1.02(0.99,1.04) |  | 0.013 | 0.065 | 1.01(1,1.03) |
| Estrogen (pg/mL) | 0.000 | 0.976 | 1(1,1) |  | 0.000 | 0.206 | 1(1,1) |
| Progesterone (ng/mL) | -0.001 | 0.959 | 1(0.96,1.04) |  | -0.004 | 0.557 | 1(0.98,1.01) |
| AFC | 0.001 | 0.868 | 1(0.99,1.01) |  | 0.008 | 0.002 | 1.01(1,1.01) |
| **Estrogen on the day before ET (High vs Low, Low=ref)** | **-0.580** | **0.000** | **0.56(0.45,0.7)** |  | **-0.162** | **0.004** | **0.85(0.76,0.95)** |
| **Progesterone on the day before ET(High vs Low, Low=ref)** | **-0.063** | **0.487** | **0.94(0.79,1.12)** |  | **-0.020** | **0.627** | **0.98(0.9,1.06)** |
| Number of embryos transferred | 0.515 | 0.000 | 1.67(1.27,2.21) |  | 0.607 | 0.000 | 1.84(1.62,2.08) |
| EM | 0.121 | 0.000 | 1.13(1.07,1.19) |  | 0.079 | 0.000 | 1.08(1.06,1.11) |
| PCOS (Yes vs No, No=ref) | 0.162 | 0.790 | 1.18(0.35,3.99) |  | -0.453 | 0.185 | 0.64(0.32,1.24) |
| Adenomyosis (Yes vs No, No=ref) | -0.369 | 0.366 | 0.69(0.3,1.51) |  | -0.203 | 0.083 | 0.82(0.65,1.03) |
| Moderate to severe intrauterine Adhesions (Yes vs No, No=ref) | 0.030 | 0.814 | 1.03(0.8,1.32) |  | -0.062 | 0.340 | 0.94(0.83,1.07) |
| Untreated hydrosalpinx Severe uterine malformations (Yes vs No, No=ref) | -0.062 | 0.000 | 0.94(0.91,0.97) |  | -0.004 | 0.541 | 1(0.98,1.01) |
| Severe uterine malformations (Yes vs No, No=ref) | -0.507 | 0.224 | 0.6(0.26,1.34) |  | -0.467 | 0.024 | 0.63(0.41,0.94) |
| Scarred uterus (Yes vs No, No=ref) | -0.067 | 0.732 | 0.94(0.64,1.36) |  | -0.288 | 0.002 | 0.75(0.62,0.9) |
| Endometritis (Yes vs No, No=ref) | 0.290 | 0.110 | 1.34(0.94,1.91) |  | 0.068 | 0.216 | 1.07(0.96,1.19) |
